# Persistent impacts of smoking on resting-state EEG in male chronic smokers and past-smokers with 20 years of abstinence

**DOI:** 10.1038/s41598-023-29547-3

**Published:** 2023-03-08

**Authors:** Hyeji Lee, Yoonji Jeon, Cheolin Yoo, HeeYoung Seon, Jiwon Park, Minho Hwang, Kwangyeol Baek, Dongil Chung

**Affiliations:** 1 Department of Biomedical Engineering, Ulsan National Institute of Science and Technology, 50 UNIST-Gil, Ulsan, 44919 South Korea; 2 School of Biomedical Convergence Engineering, Pusan National University, 49 Busandaehak-Ro, Yangsan, Gyeongsangnam-Do 50612 South Korea; 3 Department of Occupational and Environmental Medicine, Ulsan University Hospital, University of Ulsan College of Medicine, Ulsan, South Korea; 4 Present Address: Department of Psychology, The University of Edinburgh, Edinburgh, UK

**Keywords:** Addiction, Addiction

## Abstract

Smoking is a severe addictive health risk behavior and notorious for the high likelihood of relapse after attempted cessation. Such an addictive pattern in smoking has been associated with neurobiological changes in the brain. However, little is known whether the neural changes associated with chronic smoking persist after a long period of successful abstinence. To address this question, we examined resting state EEG (rsEEG) in chronic smokers who have been smoking for 20 years or more, past-smokers who have been successfully abstaining for 20 years or more, and never-smokers. Both current-smokers and past-smokers showed significantly decreased relative theta power than never-smokers, showcasing persistent effect of smoking on the brain. Other rsEEG features in alpha frequency band demonstrated distinctive patterns associated with active smoking, such that compared to never-smokers, only current-smokers, but not past-smokers, showed significantly higher relative power, EEG reactivity—power changes between eyes-closed and eyes-open conditions—, and coherence between channels. Furthermore, individual variabilities across these rsEEG biomarkers were accounted for by individuals’ self-reported smoking history and nicotine dependence in current- and past- smokers. These data suggest the persistent effect of smoking on the brain even after sustained remission for 20 years.

## Introduction

Smoking is one of the most severe addictive behaviors with health risk and the leading cause of preventable death that accounts for more than 8 million deaths each year worldwide^1^. A distinctive characteristic observed in smokers is that a large proportion of individuals who attempt to quit, particularly the ones who already attempted before^2^, is highly likely to relapse^3^. Such an addictive pattern has been conceptualized as a “cycle of spiraling dysregulation” of the brain^4^, describing a spiraling path from sensitization of dopaminergic and serotonergic neurotransmission with repeated drug intakes to their counteradaptation at withdrawal. This conceptual notion is in line with experimentally observed neurobiological changes in the brain^5–7^ that account for the reason why individuals become addicted and why they are vulnerable to relapse. This perspective was corroborated by previous studies showing that cue-induced neural responses^8–11^ and smaller brain volume in smokers are associated with a high risk of relapse^12^. A natural question that follows is whether or not smoking has long-term effects on the brain. However, it still remains elusive whether these changes in the brain of smokers are persistent even after a long period of successful abstinence. To address this issue, both the impacts of chronic smoking and of long-term abstinence need to be examined from individuals who have different histories of lifetime smoking.

Previous studies showed that electroencephalography (EEG) can be used to capture neural characteristics in psychopathology^13,14^. Among various features in EEG, the differences in spontaneous and intrinsic brain activities measured under the resting state^15^ have been found useful in dissociating smokers from never-smokers, and in characterizing smokers’ abstinence^16–18^ and craving severity^19^. Particularly, being a smoker or administrating nicotine has been typically associated with decreased EEG powers in delta or theta frequency bands^20,21^, but with increased powers in alpha band^22–24^. Note that most studies focused on the impact of smoking (acute or chronic) or a brief nicotine deprivation. It is unclear whether the EEG characteristics associated with chronic smoking are persistent over lifetime or reversible to some extent after long-term sustained remission. The aim of the current study is to examine EEG characteristics in individuals who have different smoking profiles (e.g., chronic smoking vs. successful long-term abstinence) and distinguish persistent effects of smoking from potentially recoverable ones. Based on previous reports on EEG biomarkers associated with smoking, we hypothesized that resting state EEG (rsEEG) powers measured from chronic smokers, past-smokers, and never-smokers would reveal neural changes associated with chronic smoking apart from the changes unobservable in past-smokers, as if they are recovered with successful long-term abstinence.

To examine long-term effects of smoking and abstinence, we explored EEG coherence as an additional feature in EEG. Previous studies showed that individuals with substance dependence (e.g., nicotine^25^, cannabis^26^, alcohol^27^, and heroine^28^) or behavioral addiction (e.g., gaming disorder and internet addiction^29^) demonstrate altered EEG coherence. In a recent study, Prashad et al.^26^ reported EEG coherence patterns in cannabis users, and suggested a possibility of using EEG coherence as a biomarker for substance use disorders. Winterer et al.^27^ examined EEG coherence in long-term abstinent alcohol users and showed that an increase in EEG coherence may be a trait-like (i.e., irreversible or invariant) feature for substance abuse. Based on these previous reports, we expected EEG coherence to provide a useful measure for investigating both the long-term effects of smoking and abstinence.

Neurobiologically, it is known that chronic nicotine exposure increases the number of nicotinic acetylcholine receptors (nAChR) and reduces their functional sensitivity^30,31^. In a series of studies on the neurodegenerative changes in the brain (e.g., Alzheimer’s disease), alpha reactivity—relative reduction in alpha band EEG power during eyes-open compared to eyes-closed resting state—has been suggested as a biomarker of cholinergic system integrity^32–34^. Specifically, a recent study suggested that individuals’ alpha reactivity levels are associated with their functional connectivity between the visual cortex and the nucleus basalis of Meynert (NBM)^33^, a brain region considered as the main source of cortical cholinergic innervation. Based on these findings, we expected that EEG patterns of chronic cigarette smoking may also reflect the alternations in the functional connectivity between the associated brain regions. Across various previous studies that examined the relationship between smoking and alpha rhythm EEG, the focus has been on the impact of acute nicotine administration or temporary deprivation^16,17,22,23^ and of cue-induced responses^10,35^. Here, based on previously reported neurobiological changes linked to chronic smoking, we hypothesized that alpha reactivity in addition to EEG power and coherence may reflect the effects of long-term smoking and abstinence on acetylcholine circuitry in the brain. To test this hypothesis, we took extra attention to rsEEG difference between the eye-closed vs. the eye-open conditions.

We analyzed rsEEG data from 20 chronic heavy smokers who had continued daily smoking at least for 20 years, 28 past-smokers who successfully stayed abstinent for 20 years, and 33 non-smoking controls (i.e., never-smokers) in the current study. If there exist persistent neural alterations associated with smoking, similar EEG activity patterns should be observed between smokers and past-smokers even if the latter group has been successful in staying abstinent for a very long period, whereas the patterns should be different from that of never-smokers. To further examine individual differences, we explored correlations between individuals’ EEG features (power, coherence, and alpha reactivity) and their smoking related self-report measures (e.g., cigarettes per day, nicotine dependence, and number of quit attempts).

## Results

### Smokers, past-smokers, and never-smokers were matched for all demographics but smoking-related measures

Participants across three groups were matched for age (F(2, 78) = 1.76, *P* = 0.18), gender (all male), education (χ^2^(8) = 14.37, *P* = 0.073), and income level (F(2, 78) = 0.37, *P* = 0.69; Table 1). Smokers and past-smokers were comparable in the average number of cigarettes that individuals smoked (or used to smoke) per day (t(38) = 0.90, *P* = 0.38) as well as the number of quit attempts (U = 142, *P* = 0.12). Smokers and past-smokers differed in other smoking-related characteristics. Compared with past-smokers, smokers initiated smoking at their earlier age (t(46) = − 2.95, *P* = 4.95e−03), smoked for more years (t(46) = 13.63, *P* = 9.07e−18), reported higher nicotine dependence (FTND: t(37) = 2.81, *P* = 0.0079), and exhibited higher craving at the time of the experiment (QSU-brief: t(37) = 4.46, *P* = 7.30e−05). Besides these basic demographic information and smoking-related measures, we also collected individuals’ self-report depression symptoms that may accompany their smoking history, and confirmed that all three groups showed comparable levels of depression (F(2, 78) = 0.35, *P* = 0.70). These results indicate that any group differences, if exist, can be attributed to differences in smoking status among groups rather than other comorbidities.

**Table 1:** Demographic information and smoking measures.

	Never-smoker (n = 33)	Past-smoker (n = 28)	Smoker (n = 20)	Test statistics
Age (years)	53.09 ± 4.96 (45–64)	54.64 ± 6.48 (42–69)	56.30 ± 7.12 (42–66)	F(2, 78) = 1.76 *P* = 0.18
Education^a^	3.67 ± 1.14	3.32 ± 1.12	3.00 ± 0.97	χ^2^(8) = 14.37 *P* = 0.073
Income level^b^	3.15 ± 1.25	3.14 ± 1.41	2.85 ± 1.42	F(2, 78) = 0.37 *P* = 0.69
K-BDI-II	8.63 ± 6.89 (1–27)	9.96 ± 6.50 (2–29)	8.75 ± 6.27 (0–22)	F(2, 78) = 0.35 *P* = 0.71
Age of smoking initiation**	–	19.43 ± 1.32 (17–22)	18.10 ± 1.80 (15–22)	t(46) = –2.95 *P* = 0.0049
Years smoking***	–	10.21 ± 6.43 (0.5–20)	37.80 ± 7.54 (23–50)	t(46) = 13.63 *P* = 9.07e−18
Cigarettes per day^c^	–	18.24 ± 9.05 (10–40)	20.53 ± 6.76 (9–30)	t(38) = − 0.90 *P* = 0.38
Quit attempts^c^	–	2.11 ± 2.11 (1–10)	2.15 ± 1.04 (1–5)	U = 142.00 *P* = 0.12
Abstinence duration	–	24.84 ± 4.70 years (20–36)	4.80 ± 6.28 days (2–30)	–
FTND total**^,c^	–	3.15 ± 3.17 (0–10)	5.58 ± 2.09 (3–10)	t(37) = 2.81 *P* = 0.0079
QSU-brief***^,c^	–	10.80 ± 2.73 (10–22)	21.32 ± 10.17 (10–43)	t(37) = 4.46 *P* = 7.30e−05

Means ± SDs and the range ([min–max]) of each item are reported. K-BDI-II, Korean Version of Beck Depression Inventory, Second Edition^61^; FTND, Fagerström Test for Nicotine Dependence^57,58^; QSU-brief, brief Questionnaire of Smoking Urges^59^.

^a^Average education where 1 =  ≤ middle school graduate, 2 = high school graduate, 3 = some university or community college graduate, 4 = Bachelor’s degree, 5 =  ≥ Postgraduate studies.

^b^Average household monthly income where 1 =  ≤ $1800, 2 = $1800 to 3500, 3 = $3500 to 5300, 4 = $5300 to 7000, 5 = $7000 to 8800, and 6 =  ≥ $8800.

^c^Quit attempts data are missing from ten past-smokers, and cigarettes per day data are missing from one smoker and seven past-smokers. FTND and QSU-brief data are missing from one smoker and eight past-smokers; **P < 0.01, ***P < 0.001.

### Low frequency band powers and alpha reactivity are associated with individuals’ smoking status and habits

**Figure 1:**
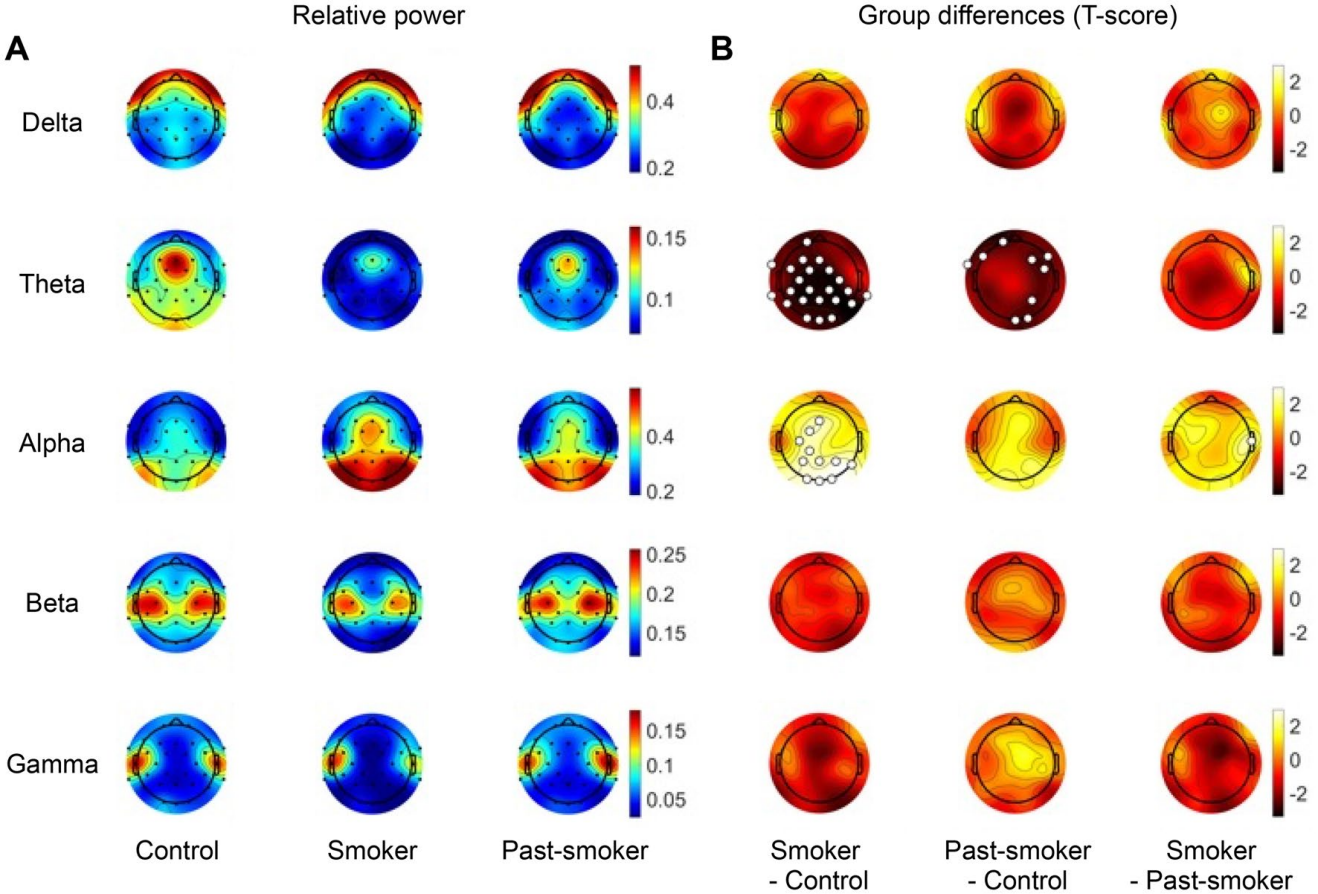
Relative EEG band power (eyes-closed condition). (**A**) Each row illustrates the average relative band powers calculated in each group. (**B**) Statistical group differences in relative band powers were observed in theta and alpha bands, particularly between the Smoker and the Control groups. Specifically, never-smoker controls showed larger relative power in theta band compared to the smoker and past-smoker groups, while smaller power in alpha band than the smoker group. The channels that showed a significant group difference are marked with larger unfilled markers (*P*_FDR_ < 0.05).

To test whether EEG powers are associated with individuals’ chronic smoking and abstinence, relative spectral powers were calculated in each frequency band for each group of individuals. In the eyes-open condition, there was no significant group difference in EEG relative band powers. On the contrary, for the eyes-closed condition, significant group differences were found in theta and alpha bands (Kruskal–Wallis test, false discovery rate adjusted *P (P*_*FDR*_*)* < 0.05; Fig. 1). Specifically, relative theta band power was significantly lower in smokers compared with never-smokers in a widespread area including the channels from the frontal, central, parietal, and occipital regions (Fp1, F3, Fz, FT9, FC1, FC2, FC5, C3, C4, TP9, CP1, CP2, CP5, CP6, P3, P4, P7, P8, Pz, O1, O2, and Oz; *P* < 0.05, post-hoc Tukey’s test). Past-smokers also showed diminished relative theta band power, such that their theta band powers from the frontal and occipital regions (Fp1, F4, F7, F8, FT9, FC6, P4, O2, and Oz; *P* < 0.05 in post-hoc Tukey’s test) were significantly lower than that of never-smokers. No significant theta power differences were observed between smokers and past-smokers.

In relative alpha band power, significant group differences were largely observed between the smoker and never-smoker groups. Smokers exhibited higher alpha power compared with never-smokers in the central and occipital regions (Fz, FC1, C3, CP1, P3, P4, P8, Pz, O1, O2, and Oz; *P* < 0.05, post-hoc Tukey’s test). Alpha band power in past-smokers was comparable with that of both the other two groups in all regions, except in T8 channel where smokers showed higher power than past-smokers (*P* < 0.05, post-hoc Tukey’s test).

**Figure 2:**
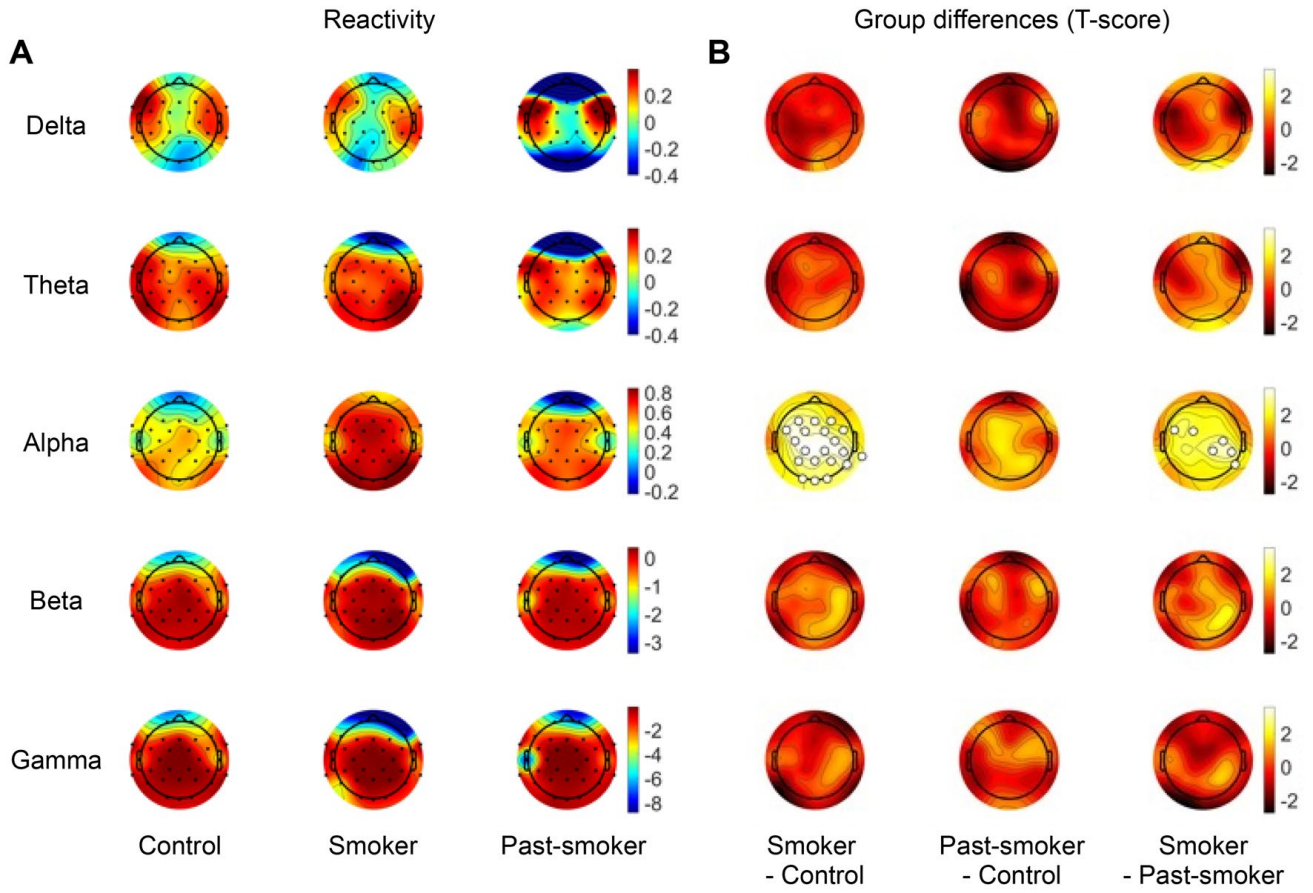
EEG reactivity between the eyes-closed and the eyes-open conditions. (**A**) Each row illustrates average EEG reactivity measures calculated in each group, for each frequency band. (**B**) Statistical group differences in EEG reactivity were observed in alpha band. Particularly, smokers showed larger alpha reactivity compared with past-smokers or non-smoking controls. The channels that showed a significant group difference are marked with larger unfilled markers (*P*_FDR_ < 0.05).

It is worth noting that group differences in low frequency powers (theta, alpha bands) associated with smoking status were only observed from the eyes-closed condition, and that such a discrepancy mirrors previous reports regarding alpha reactivity—power reduction at eyes-open compared to eyes-closed condition within a specific frequency band^32–34^. To directly test our hypothesis about its relevance to chronic smoking, we examined group differences in alpha reactivity. As hypothesized, smokers’ alpha reactivity was significantly higher than that of never-smokers and past-smokers (Kruskal–Wallis test: *P*_*FDR*_ < 0.05; Fig. 2). Post-hoc Tukey’s test revealed that smokers showed larger alpha reactivity than never-smokers in a broad area (FP1, F3, F4, F7, Fz, FC1, FC2, FC5, FC6, C3, C4, CP1, CP2, CP6, TP1, P3, P4, P8, Pz, O1, O2, and Oz; *P* < 0.05, post-hoc Tukey’s test). Moreover, a part of the increased alpha reactivity in smokers, observed from the central and parietal regions (FC5, FC1, C4, CP6, CP2, and P8; *P* < 0.05, post-hoc Tukey’s test), was significantly higher than that in past-smokers. For completeness, we also calculated EEG reactivity in other frequency bands. However, there was no significant reactivity difference in other frequency bands between groups.

We further examined whether these features in low frequency bands are associated with individuals’ smoking-related characteristics other than their smoking status. Across the two smoker groups (smokers and past-smokers), the amount of cigarette consumption, nicotine dependence, and the number of quit attempts were significantly correlated with the relative powers in the theta frequency band (Fig. 4). Particularly, individuals who smoked more number of cigarettes per day (Pearson’s correlation coefficient r = − 0.31, bootstrapped *P* = 0.033) or reported higher nicotine dependence (FTND: r = − 0.40, bootstrapped *P* = 0.002) showed lower theta band power, while individuals who had more attempts to quit smoking showed higher theta band power (r = 0.43, bootstrapped *P* = 0.033). In contrast, there was no significant association between relative alpha power or alpha reactivity and individuals’ smoking-related self-report measures. These results indicate that theta band powers measured from eyes-closed rsEEG not only reflect the persistent effect of smoking across current- and past-smokers, but also capture detailed individual differences in their nicotine dependence severity and smoking behaviors independent of their recency.

Note that nicotine dependence (measured by FTND) was significantly different between current- and past- smokers (Table 1), and thus, the correlation between the dependence and theta power (Fig. 4A) could potentially reflect the group difference. When each group was examined separately, past-smokers still showed a significant association between their nicotine dependence and theta power (r = − 0.47, bootstrapped *P* = 0.011). In current-smokers, the association was trending in the same direction, but statistically not significant (r = − 0.28, bootstrapped *P* = 0.29). These results suggest that individuals’ theta powers reflect both their nicotine dependence and smoking status.

### Alpha coherence is associated with individuals’ smoking status and habits

**Figure 3:**
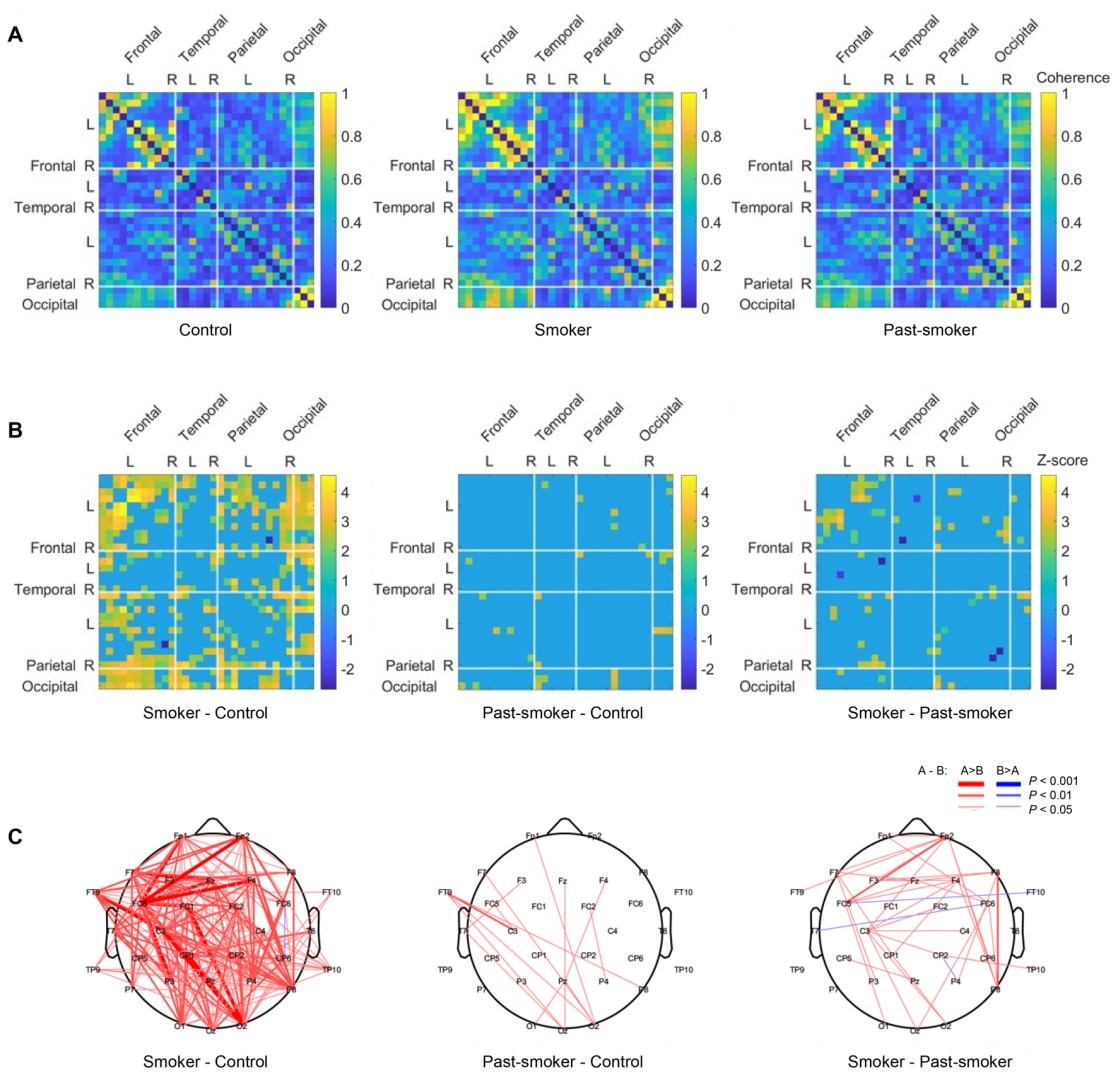
Group differences in alpha band coherence. (**A**) Alpha band coherence between EEG channels was calculated in each group. (**B**) Significant group differences in alpha band coherence were observed most distinctively between the Smoker and the Control groups. (**C**) For an illustrative purpose, all significant group differences in inter-channel coherences are depicted with three different thickness levels (*P*_FDR_ < 0.05, 0.01, and 0.001).

To test whether EEG coherence is associated with smoking as observed in other substance use disorders, we examined group differences in alpha band coherence across every pairs of channels (Fig. 3A). As in the aforementioned patterns of EEG powers, significant group differences were observed from the coherence during the eyes-closed condition (Kruskal–Wallis test: *P*_*FDR*_ < 0.05; Fig. 3). Specifically, smokers had significantly higher alpha band coherence compared to never-smokers, primarily at the channel pairs among the left frontal, left parietal, and occipital regions (Fig. 3B,C). Among the alpha band coherence in smokers, coherence between the left and right frontal regions was higher than corresponding coherence patterns in past-smokers (Fig. 3B,C). Coherence in past-smokers was largely comparable with that in never-smokers, except for a few pairs of channels between the left frontal and occipital regions (Fig. 3B,C). No significant group difference in alpha band coherence was found in the EEG recordings during the eye-open condition. For completeness, we also examined EEG coherence in other frequency bands for the eye-closed and eye-open conditions, albeit no significant group difference was observed.

**Figure 4:**
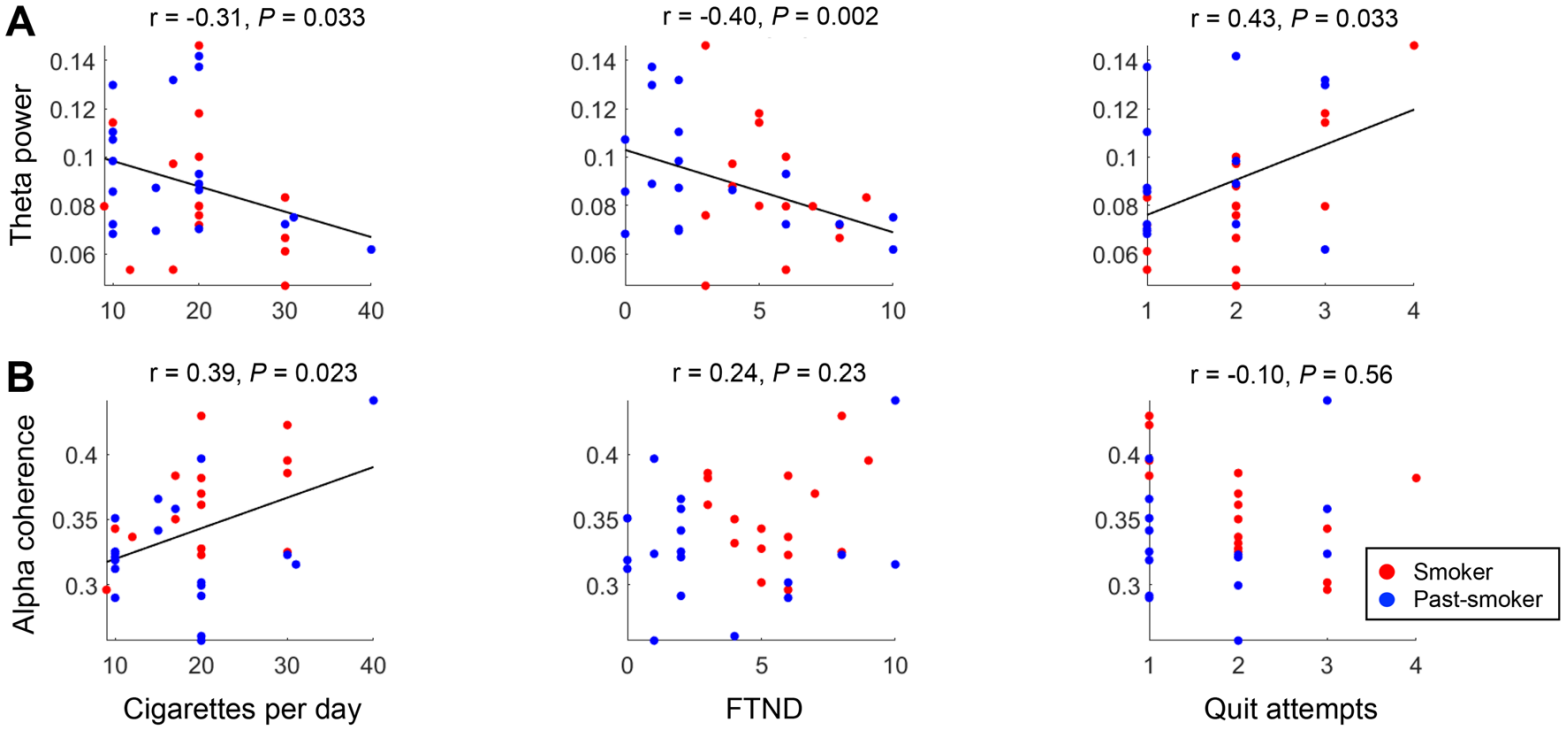
Correlations between EEG features and individuals’ smoking-related characteristics. Pearson’s correlation coefficients were calculated to examine the association between EEG characteristics (relative EEG band powers and coherence) and individuals’ smoking history measures (cigarettes per day, FTND, and number of quit attempts). (**A**) Across smokers and past-smokers, individuals who have smoked more (or had smoked more in the past for past-smokers) showed lower theta band power, (**B**) but higher alpha coherence. (**A**) In addition, individuals who self-reported higher nicotine dependence (FTND score) showed lower theta band power, and who had more quit attempts showed higher theta power. Each dot represents an individual participant, and the color-coded lines are the regression line between the corresponding measures.

We further examined correlations between individuals’ coherences and their smoking-related measures to test whether the increased coherences are associated with individuals’ smoking history and/or severity. Regardless of smoking status, individuals who smoked more number of cigarettes per day showed significantly higher alpha coherence (r = 0.39, bootstrapped *P* = 0.023; Fig. 4B), evidencing for the severity effect on individuals’ EEG. On the contrary, there was no significant evidence suggesting the associations between EEG coherence and other self-report measures (nicotine dependence, quit attempts, or years of smoking). Consistent with previous findings in individuals with problematic substance uses (e.g., alcohol)^27^, these coherence results suggest that chronic and intensive smoking may alter the brain’s functional connectivity, which in turn increases coherences in the rsEEG.

## Discussion

The current study examined three groups of individuals with different smoking profiles and investigated the persistent (or nonpersistent) effects of smoking on the brain. Both current- and past- smokers showed lower theta band power in rsEEG than non-smoking controls during eye-closed condition, demonstrating that impacts of smoking persist even after 20 or more years of abstinence. On the contrary, EEG features in alpha frequency band (i.e., higher alpha band power, higher alpha reactivity, and higher alpha band coherence) distinguished smokers from never-smokers; these alpha band differences were not observed between past- and never- smokers. The EEG patterns observed in long-term abstainers suggest that smoking-related EEG alterations in alpha frequency band are recoverable to some extent after a sufficient period of abstinence. In addition to the group differences, individuals’ self-report smoking behaviors and dependence accounted for the individual differences in the theta band powers and alpha band coherence across current- and past- smokers. These results showcase neural impacts of smoking on rsEEG that not only reflect individuals’ current state (e.g., nicotine dependence) but also the history of smoking (e.g., daily cigarette consumption).

Supporting the widely accepted view of addiction as a brain disease^5,36^, long-term exposure to addictive substances, regardless of their legality, is known to trigger functional and structural changes in the brain^37^. Based on these neurobiological changes, pharmacological targeting^38^ and brain stimulation^39^ have been suggested as plausible treatments for reversing the impacts of addiction on the brain. To date, however, previous studies focused on either the impacts of brief abstinence or that of acute nicotine consumption^40^, and it still has not been directly examined whether the neural traces of individuals’ smoking history in the brain *ever* disappear even after a long period of successful abstinence (c.f., see Karama et al.^41^ for the impacts of smoking on cortical thickness suggested to be reversible with long-term cessation). The current study addresses this gap by examining past-smokers who smoked daily for at least half a year and stayed abstinent for 20 years or longer. Moreover, smokers were recruited from a group behavior therapy for smoking cessation, and all the participants in the smoker group were mandated to be free from the recent influence of nicotine at the time of experiment. These settings matched sobriety of the two smoking groups. Thus, the differences we report cannot be interpreted as results of instant satiety in the smoker group, but rather as patterns distinguishing the consequence of chronic smoking vs. long-term complete abstinence.

Previous studies reported that nicotine-deprived smokers compared to never-smokers or to non-deprived smokers showed reduced power in low frequency bands (i.e., delta and theta)^20,24^. In contrast to these results, another study that specifically examined rsEEG of non-deprived smokers observed a similar pattern of reduced low frequency power compared to that of non-daily smokers^42^. This finding suggested that the changes in rsEEG might not simply be reflecting a state of withdrawal, but also associated with categorical smoking status (e.g., daily smoker, nondaily smoker). As noted above, all participants in the current study were abstinent at the time of the experiment, and thus any observed differences could be attributed to individuals’ smoking history (e.g., severity, quit attempts, dependence) and status (e.g., current-, past-, and non- smoker). The persistent effect of smoking observed both in current- and past- smokers in the present study was consistent with previous results, such that theta band power in the frontal and occipital regions was lower in smokers than never-smokers. Beyond this group difference, these patterns were more distinctive in individuals who have (or had) smoked more number of cigarettes per day, had less quit attempts, and had higher nicotine dependence. These results suggest that reduced low frequency power is a neural marker reflecting both individuals’ smoking history and dependency, and that the ‘scar’ remains even after a very long abstinence.

In a neurobiological perspective, long-term nicotine exposure is known to induce upregulation of nicotinic acetylcholine receptors and their desensitization^31,43,44^. Such neurobiological impacts of smoking may underlie the changes in functional connectivity between cortical regions and the NBM^33^, and in turn changes in cortical activities and EEG signals (e.g., alpha reactivity)^45^. Previous reports pointed out that part of these chronic changes may be recoverable. Particularly, it was reported that nAChR availability returns to the level comparable to non-smokers with 6–12 weeks of abstinence^46^. Complementary to these results, current-smokers exhibited heightened alpha reactivity compared to never- or past- smokers, whereas, as observed from the power spectrum patterns, past-smokers showed alpha reactivity pattern comparable to never-smokers. These results may indicate that neural changes induced by chronic smoking are recovered through a long period of abstinence. Alternatively, the observed difference among groups may be reflecting transient changes induced by a short period of abstinence (5 days on average) in current-smokers rather than by chronic smoking per se.^46^ Future study may directly collect both EEG and metabolic imaging (e.g., single-photon emission computed tomography; SPECT) to confirm the neural mechanism of alpha reactivity in relation to plastic change in the acetylcholine circuitry.

Relatively little is known about the impacts of smoking on EEG coherence^40^. One previous study, while the sample size was small, reported a consistent pattern in alpha coherence, such that smokers showed higher coherence than age-matched never-smokers^47^. Our data replicate and expand this result. Current-smokers showed higher alpha coherence than never-smokers across the whole brain, and moreover, past-smokers still showed higher coherence compared with never-smokers between the occipital and left frontal/temporal regions. Most interestingly, such an alteration in alpha coherence was associated with the number of cigarettes individuals smoked a day, regardless of their smoking status; individuals who smoked the most cigarettes a day, even after 20 years of abstinence, showed the highest alpha coherence. These results support that not only can EEG coherence be used as a biomarker detecting substance use disorder (e.g., cannabis use^26^, internet addiction^29^), but also as a measure reflecting a trace of long-term substance uses (e.g., alcohol use^27^).

The current study has the following limitations. First, it is a cross-sectional study and thus, we cannot confirm whether the observed neural patterns (e.g., lower theta power) are indeed consequences of chronic substance use (or abstinence) or individuals’ traits that should get credits for making them more (or less) prone to substance abuse. Future study with longitudinal tracking of individuals’ substance use history in conjunction with their neural data may provide further insights about causal roles of their brain’s characteristics in initiation of substance dependence^48^ as well as in sustained remission^49^. Second, the current study only includes data from male participants, and thus leaves it untested whether the same EEG features can be used in characterizing female smokers. Previous studies suggested that female smokers have different smoking behavior^50^ and moreover, different nicotine metabolism^51^. Given these reports, it is reasonable to expect that female smokers may show different neural signatures along their chronic smoking and abstinence. Direct investigation about and comparison between smokers of all genders would further expand our understanding of why there is a higher percentage of male smokers in general^52^ and why some individuals succeed better at quitting. Third, current-smokers who participated in the current study were all in an unsatiated state. As noted above, this inclusion criterion was set intentionally to investigate the effects of smoking history and status, rather than the effect of acute nicotine satiety. However, we cannot overlook that the same nicotine abstinence may have differential impacts on different groups; acute abstinence of smokers is likely to trigger neural and behavioral withdrawal symptoms, which must not be present in abstinent past-smokers due to the significant duration of abstinence. Future studies should examine the impact of acute nicotine satiety (or deprivation) above and beyond the long-term effects of smoking (or abstinence).

To conclude, we investigated how chronic smoking and long-term sustained remission affect the resting state EEG activity. Our data show that the EEG biomarkers in chronic smokers can also be observed at a significant level in past-smokers with more than 20 years of sustained remission, which suggests long-lasting and potentially irreversible impacts of smoking. It has been already well known that smokers who attempt to quit are highly likely to relapse and there are various factors associated with their failures^2,3^. Most of these risk factors (e.g., nicotine dependence, social environment) predicted relapse within the first few months of quitting, but failed to predict why some individuals still relapse after a few years of abstinence^53^. Our findings provide a neural explanation why individuals who ever initiated smoking in a lifetime may have changes in their brain and show different neural responses to perceptual^11^ and social information^54^, and also might be at constant risk of further problem behavior^55^.

## Methods

### Participants

Twenty-five chronic smokers, 31 past-smokers, and 38 never-smokers were recruited for the current study. Chronic smokers were recruited from a one-week group behavior therapy for smoking cessation, organized at Ulsan University Hospital, and all the smokers have been smoking daily for at least 20 years (total cigarette consumption > 18 pack-years). The exclusion criteria included neurological or psychiatric disorder, traumatic brain injury, and current use of psychoactive substances other than nicotine, and were initially assessed at the sign-up interview for the current study. Only three out of 25 smokers who met the inclusion criteria were female, which reflects a large difference in smoking rate between male vs. female in South Korea^56^. Due to the limited number of female smoker subjects, we decided to focus our investigation to male population only and left it as a limitation in the present study (see “Discussion”). Two additional smokers were excluded due to large EEG artifacts (see “EEG analyses” for artifact definition). At study intake, all smoker participants were abstinent for at least 24 h and only five participants smoked 6.20 ± 5.63 cigarettes in last 48 h. Before the EEG measurement, participants’ abstinence was confirmed by the exhaled carbon monoxide (eCO) level of 3 ppm or below (Micro Smokerlyzer, Bedfont Scientific Ltd., Maidstone, Kent, UK; BMC-2000, Senko International Inc., Osan-si, Gyeonggi-do, Republic of Korea). Never-smokers and past-smokers were recruited from local community via online advertisements. Never-smokers were strictly defined as individuals who smoked < 5 cigarettes in their lifetime and had not smoked in last month. To examine the effect of chronic smoking that persist after a long period of abstinence, we set inclusion criteria for the past-smoker group as following: (1) regularly smoked 10 or more cigarettes per day at least for 6 months, and (2) has been ceased smoking for 20 years or more. As in the smoker group, none of past- or never- smokers met the following exclusion criteria: history of neurological disorder, psychiatric disorder, or traumatic brain injury, and current use of psychoactive substances. Past- and never- smokers were also confirmed that they showed eCO level of either 0 or 1 ppm. Three past-smokers and five never-smokers were excluded due to extreme EEG artifacts (see “EEG analyses” for artifact definition). After applying aforementioned exclusion/inclusion criteria, final sample included 20 smokers (age = 56.30 ± 6.93), 28 past-smokers (age = 54.64 ± 6.36), and 33 never-smokers (age = 53.09 ± 4.88). See Table 1 for additional demographic information and smoking histories. The current study and all the protocols were approved by the Institutional Review Board (IRB) of Ulsan University Hospital and Ulsan National Institute of Science and Technology (UNIST) (UNISTIRB-19-45-A). All research was performed in accordance with relevant guidelines and regulations, and all participants provided written informed consent following an explanation of study procedures.

### Smoking history and clinical measures

Smokers and past-smokers completed a battery of self-report measures, assessing their smoking history, nicotine dependence, and craving for nicotine. Nicotine dependence was measured using the Fagerström Test for Nicotine Dependence (FTND)^57,58^, and craving for nicotine was measured using a brief, 10-item version of the Questionnaire of Smoking Urges (QSU-brief)^59^. Given a tight association between smoking cessation attempts and depression^60^, individuals’ depression symptoms were also measured using Korean version of Beck Depression Inventory, second edition (K-BDI-II)^61^.

### EEG acquisition

The electroencephalogram (EEG) was digitized at a sampling frequency of 500 Hz, amplified using the actiCHamp system (Brain Products GmbH, Germany), and recorded from 31 Ag/AgCl electrodes mounted in a cap (actiCap, Brain Products GmbH, Germany). We used the Fz electrode as a reference and the FPz as a ground. Impedances of all electrodes were maintained at ≤ 5 kΩ. Resting state EEG was recorded for three minutes during eyes-open and three minutes during eyes-closed conditions. During the eyes-open condition, participants were instructed to fixate their eyes at a crosshair located at the center of the monitor.

### EEG analyses

EEG data analysis was conducted using EEGLAB version 2020.0 (Swartz Center for Computational Neuroscience, University of California at San Diego, CA) and MATLAB R2019b (Mathworks Ltd., Natick, MA). Raw EEG data were first filtered using a bandpass filter between 1 and 50 Hz. We utilized ICA algorithm in EEGLAB to identify and correct for eye blinking, muscle activity, line noise, and motion-related artifacts. Then, EEG data were re-referenced using the Common Average Reference (CAR). The noise-free periods in the EEG recordings were selected by visual inspection and sliced into two-second epochs. Fast Fourier Transform (FFT) was applied to preprocessed EEG to calculate signal power of EEG, i.e., power spectra (µV^2^). Spectral powers of the epochs were calculated for five separate frequency bands: Delta (1–4 Hz), Theta (4–8 Hz), Alpha (8–13 Hz), Beta (13–30 Hz), and Gamma (30–50 Hz).

From the initial participant pool, 2 smokers, 3 past-smokers, and 5 never-smokers were excluded from further analysis due to excessive EEG artifacts (> 75 μV throughout the entire EEG or EEG spectral power deviated > 3 SD from the group mean in each condition). In the remaining participants, EEG data with the spectral power deviated > 3 SD from the group mean in a single condition (i.e., eyes-open or eyes-closed) were only excluded from the statistical analysis for the corresponding condition: 5 smokers, 6 past-smokers, and 3 never-smokers.

To examine impacts of smoking status among groups, we calculated relative spectral powers, alpha reactivity, and alpha band coherence. Relative spectral powers were calculated in the following steps. First, FFT was applied to each of two-second epochs, and their power spectra were calculated. Second, absolute spectral powers of five separate frequency bands (i.e., delta, theta, alpha, beta, and gamma) were calculated for each epoch. Third, to obtain average band powers for each individual, absolute spectral band powers were averaged across epochs. Fourth, relative spectral powers of each frequency band were defined as a ratio between the absolute power of the corresponding frequency band and the total sum of absolute power from 1 to 50 Hz. To examine alpha band reactivity, we calculated EEG band power changes between eyes-closed and eyes-open conditions as follows^33^:

$$EEG reactivity = \frac{{EEG band power}_{eyes-closed}- {EEG band power}_{eyes-open}}{{EEG band power}_{eyes-closed}}$$

According to this definition, individuals who show larger alpha power difference between eyes- open and closed conditions have larger EEG reactivity.

The alpha band coherence was defined as the magnitude-squared coherence of the two signals using Welch's mean-corrected periodogram^62^. Given the two stationary signals *x* and *y*, the magnitude-squared coherence is defined as follows:

$${C}_{xy}\left(f\right)=\frac{{\left|{P}_{xy}(f)\right|}^{2}}{{P}_{xx}\left(f\right) {P}_{yy}(f)}$$

where *P*_*xx*_*(f)* and *P*_*yy*_*(f)* are the power spectral density of signals *x* and *y*, respectively, and *P*_*xy*_*(f)* is the cross power spectral density of the two signals*.* The alpha band coherence was calculated between all pairs of EEG channels in alpha frequency band.

### Statistical analyses

We conducted one-way analysis of variance (ANOVA) with post-hoc Tukey’s tests for comparing age, income levels, and self-report depression levels (K-BDI-II) among the groups, and used the chi-squared test for comparing education levels among the groups. To test whether smokers and past-smokers differed on their smoking behaviors and self-report attitude against nicotine (dependence and craving), we used independent sample t-tests and compared age of smoking initiation, years of smoking, average number of cigarettes smoked per day, nicotine dependence (FTND total score), and nicotine craving (QSU-brief score). Mann–Whitney U test was used in comparing number of quit attempts between smokers and past-smokers, because the data violated assumptions of normality. Group differences in EEG band powers, alpha reactivity, and alpha band coherence were evaluated using Kruskal–Wallis test, a non-parametric test equivalent to one-way ANOVA. In all tests comparing EEG features, significance level was set at p-value < 0.05 after controlling for False Discovery Rate (FDR) across 31 channels, and post-hoc Tukey’s tests were conducted for comparison between groups. To further examine the associations between EEG features and individuals’ smoking-related characteristics, we first calculated the average of each EEG feature for each individual and used them as an individual’s representative neural measure. Specifically for relative theta power, alpha power, and alpha band reactivity, we averaged the calculated feature measures across all 31 channels for each individual. For alpha band coherence, we averaged the lower triangle of the coherence matrix, given that coherence values are symmetric between the lower and upper triangles. We evaluated Pearson’s correlations between EEG features and individuals’ self-reported measures related to smoking (e.g., FTND, QSU-brief, number of cigarettes smoked per day). Nonparametric bootstrapping method was used to compute confidence intervals (number of the bootstrapped samples = 10,000).

## Data Availability

The datasets generated during and analyzed during the current study are available at https://github.com/dongilchung/rsEEG-smoker.
